# Clinical development of phosphatidylinositol 3-kinase inhibitors for non-Hodgkin lymphoma

**DOI:** 10.1186/2050-7771-1-30

**Published:** 2013-11-15

**Authors:** Xiaosheng Fang, Xiangxiang Zhou, Xin Wang

**Affiliations:** 1 Department of Hematology, Shandong Provincial Hospital affiliated to Shandong University, 324 Jingwu Road, Jinan, Shandong 250021, P.R. China; 2 Department of Diagnostics, Shandong University School of Medicine, Jinan, Shandong 250012, P.R. China

**Keywords:** PI3K inhibitors, NHL, Clinical development

## Abstract

Phosphatidylinositol 3-kinase (PI3K)/Akt/mammalian target of rapamycin (mTOR) signaling pathway is extensively explored in cancers. It functions as an important regulator of cell growth, survival and metabolism. Activation of this pathway also predicts poor prognosis in numerous human malignancies. Drugs targeting this signaling pathway have been developed and have shown preliminary clinical activity. Accumulating evidence has highlighted the important role of PI3K in non-Hodgkin lymphoma (NHL), especially in the disease initiation and progression. Therapeutic functions of PI3K inhibitors in NHL have been demonstrated both in vivo and in vitro. This review will summarize recent advances in the activation of PI3K signaling in different types of NHL and the applications of PI3K inhibitors in NHL treatment.

## Introduction

The PI3K/Akt/mTOR pathway plays a critical role in regulating cancer cell growth, survival, motility and metabolism [1]. Phosphatidylinositol 3-kinase (PI3K) is a critical element in this signaling, it is activated in a wide range of human neoplasms and associated with poor outcomes [2,3]. Our previous studies have demonstrated that down regulation of heat shock protein 70 (HSP70) contributed to the increased sensitivity of Burkitt lymphoma (BL) cells to chemotherapy through blocking this pathway [4]. Targeted inhibitors for PI3K signaling are opening a new paradigm in cancer treatment. Activation of this pathway was identified in different types of NHL [5]. A number of PI3K inhibitors have been developed and displayed preliminary clinical activities in NHL treatment.

### The PI3K signaling pathway in cancer

**Figure 1:**
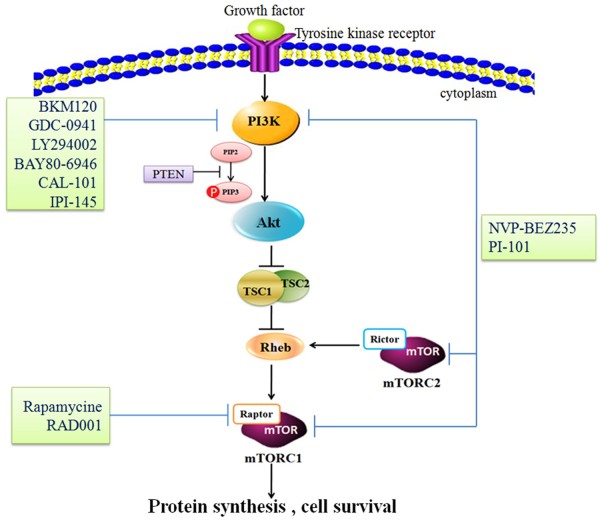
**The PI3K/Akt/mTOR pathway and relative inhibitors in NHL.** Once RTKs binding to the growth factors, the PI3K signaling pathway is triggered. Activated PI3K could phosphorylate PI (4,5) P2 (PIP2) to produce PI(3,4,5)P3 (PIP3). This process is negatively regulated by PTEN. Akt inhibits the Rheb GTPase activity of TSC1/TSC2 dimer by phosphorylating TSC2. Then activated Rheb stimulates mTOR to phosphorylate the p70S6 and 4E-binding protein (4EBP-1), resulting in dysregulation of protein synthesis and cell survival. On the other hand, mTORC2, another type of mTOR complex, could phosphorylate Akt and promote the complete activation of it.

The PI3K signaling pathway is triggered by activation of receptor tyrosine kinase (RTK) in cell membrane. After binding to the growth factors, the intracellular domain of RTK is phosphorylated, and PI3K is activated (Figure 1). There are three classes (I, II, III) of PI3Ks, with class I PI3Ks as the most studied in human cancer [6]. Activated PI3K phosphorylates PI(4,5)P2 (PIP2) to produce PI(3,4,5)P3 (PIP3). The tumor suppressor phosphatase and tensin homolog (PTEN) deleted on chromosome ten could negatively regulate this process through dephosphorylating PIP3. Activated PIP3 could prompt the phosphorylation of Akt and further stimulate the Akt­mediated activation of downstream targets, including the Bcl-2 family members, Mdm2 and tuberous sclerosis complex 2 (TSC2) [7]. Activated Akt inhibits the Rheb GTPase activity of TSC1/2 complex through phosphorylating TSC2. Then the activated Rheb promotes mTOR complex 1 (mTORC1) to phosphorylate p70S6 and 4E binding protein1 (4EBP1), resulting in dysregulation of protein synthesis and cell survival [8]. On the other hand, mTORC2, another type of mTOR complex, could phosphorylate Akt on serine 473 and facilitate its complete activation [9].

The PI3K/Akt/mTOR pathway is constitutively activated in human cancers and is critical for tumor progression and chemo-resistance [10]. Alterations of several components in this pathway have been identified in numerous tumors [11]. Mutation of PI3KA was most commonly recognized in breast, colorectal and endometrial cancers [12]. And the alteration of Akt was found in gastric, pancreatic and ovarian cancers. These alterations promoted the development of PI3K pathway-specific inhibitors [7]. Several PI3K pathway inhibitors have been developed and are being evaluated in preclinical or clinical studies. As PI3K/Akt/mTOR pathway plays a key role in the proliferation and survival of lymphoma cell, various inhibitors targeting this pathway have been studied in different types of NHL (Table 1). In spite of preclinical studies, several PI3K inhibitors for NHL treatment are currently undergoing various stages of clinical trials (Table 2) [13]. Here we will focus on the clinical development of PI3K inhibitors for NHL.

**Table 1:** Different PI3K pathway inhibitors in NHL

**Inhibitors**	**Targets**	**Lymphoma type**	**References**
NVP-BEZ235	Dual PI3K and mTOR	FL, DLBCL, MCL,T-ALL	[14-17]
CAL-101	Isoform-specific PI3K(δ)	CLL, MCL	[18,19]
NVP-BKM120	Pan-isoform PI3K	B-CLL, DLBCL	[20,21]
LY294002	Pan-isoform PI3K	DLBCL, MCL, BL, T-ALL	[22-25]
GDC-0941	Pan-isoform PI3K	MCL, T-cell lymphoma	[26,27]
RAD001	mTOR	MCL	[28]
PI-103	Dual PI3K and mTOR	BL, T-ALL	[25,29]

**Table 2:** PI3K pathway inhibitors in clinical development for NHL treatment

**Drugs**	**Target**	**Clinical trial phase**	**NHL type**	**NCT no.**
**Pan-isoform PI3K inhibitor**				
NVP-BKM120	Class I PI3K	II (recruiting)	Relapsed and refractory NHL (DLBCL, MCL or FL)	NCT01693614
GDC-0941	Class I PI3K	I (completed)	NHL	NCT00876122
BAY80-6946	Class I PI3K	II (recruiting)	Relapsed, indolent or aggressive NHL (FL, MALT lymphoma, CLL, LPL/WM, DLBCL, MCL, peripheral T-cell lymphoma, ALCL)	NCT01660451
**Isoform specific PI3K inhibitor**				
CAL-101 (GS-1101)	p110δ	III (recruiting)	Previously treated indolent NHL (FL, SLL, LPL/WM, MZL)	NCT01732913
		I/II (recruiting)	Previously treated low-grade (indolent) B-cell NHL (FL, SLL, MZL)	NCT01306643
IPI-145	p110γ/δ	II (recruiting)	Refractory indolent NHL (FL, MZL, or SLL)	NCT01882803
		I (recruiting)	Relapsed/refractory hematologic malignancies (NHL, CLL, T-cell lymphoma)	NCT01871675
**Dual PI3K/mTOR inhibitor**				
NVP-BEZ235	PI3K/mTOR	I (recruiting)	relapsed or refractory acute leukemia (ALL, AML, CML-BP)	NCT01756118
**Allosteric mTOR inhibitor**				
RAD001	mTOR	I/II (recruiting)	NHL	NCT01567475
		I/II (recruiting)	relapsed or refractory NHL or HL	NCT01075321
		II (completed)	Relapsed or Refractory DLBCL	NCT00869999

MALT, mucosa-associated lymphoid tissue; LPL/WM, lymphoplasmacytic lymphoma/Waldenström macroglobulinemia; SLL, small lymphocytic lymphoma; MZL, marginal zone lymphoma; AML, acute myeloid leukemia; CML-BP, chronic myeloid leukemia in blastic phase.

### PI3K inhibitors in follicular lymphoma

Follicular lymphoma (FL) is one of the most common types of indolent NHL. In spite of its indolent phase, about 25%-60% of them eventually transform into diffuse large cell lymphoma (DLBCL), a type of aggressive lymphoma. Combination therapy included rituximab cannot significantly decline the relapse rate of FL [14]. Therefore, novel effective therapeutic agents are urgently needed to improve the outcomes of FL patients.

Gulmann C et al. demonstrated the activation of PI3K/Akt/mTOR pathway in FL by proteomic analysis [30]. They provided evidence that activation and phosphorylation of PI3K as well as its downstream effectors, including Akt, mTOR, and S6K, were found in FL. Recently, a PI3K/mTOR module was reported to mediate the invasion and angiogenesis of FL, which further confirmed its potential use in anti-invasive of FL [31]. NVP-BEZ235, a dual PI3K and mTOR inhibitor, was indicated to be effective in inhibiting FL cell proliferation [14]. Proliferation of FL cell line was substantially inhibited by NVP-BEZ235, activation level of caspase-3 increased by 1.6 to 2 fold in NVP-BEZ235-treated cells compared to that treated with vehicle alone [14]. In addition, anti-tumor function and the therapeutic potential of NVP-BEZ235 were also identified in other human malignancies, such as T-cell acute lymphoblastic leukemia (T-ALL), colorectal and lung cancer [15,32,33].

### The roles in chronic lymphocytic leukemia

Chronic lymphocytic leukemia (CLL) is the most common type of adult leukemia in the western world, with 15,000 new cases and approximately 4,500 deaths per year [34]. It is characterized by accumulation of malignant B cells in the blood, bone marrow and secondary lymphoid tissues [35]. Novel targeted agents and potential therapeutic options have been provided recently [36,37].

Consistent expressions of PI3K-δ were found in both primary CLL cells and normal B cells, but the CLL cells represented a statistically higher intrinsic PI3K activity compared to normal B cells [18,38]. CAL-101 (GS-1101) is a specific inhibitor of PI3K-δ isoform. It could prevent the proliferation and induce apoptosis of CLL cells through disrupting multiple external pathways. Activation of Akt, and secretion of cytokines and chemokines were inhibited by CAL-101 in both vitro and vivo [18,39,40]. B cells from 16 CLL patients were treated with CAL-101 at different concentrations for 48 hours [18]. The results showed that CAL-101 promoted CLL cells apoptosis in a dose- and time-dependent pattern.

Coutre et al. have reported a phase I study using CAL-101 as a single agent for relapsed/refractory CLL patients [41]. About 80% of them achieved >50% reduction in the size of lymph node and spleen. On the contrary, approximately >50% increase in lymphocytosis of peripheral blood occurred in 58% patients. This trial also provided evidence of limited toxicity of CAL-101 in CLL treatment [41]. A phase I study of CAL-101 in combination with rituximab or bendamustine in 20 patients with relapsed/refractory B-cell malignancies (indolent B-cell NHL n = 12, CLL n = 8) reached the same conclusion as well. The main adverse effects, Grade 3 neutropenia and thrombocytopenia, were found in 22% of patients receiving bendamustine plus CAL-101. Additionally, the peripheral lymphocyte counts were stable or decreased in 8/8 CLL patients after combination treatment [42].

NVP-BKM120 is an orally available pan class I inhibitor of PI3K. It was reported to inhibit the phosphorylation of Akt in primary B-CLL lymphocytes and further inhibit the PI3K signaling [20]. NVP-BKM120 also contributed to the concomitant Mcl-1 downregulation and Bim induction though regulating the Akt/FoxO3a/Bim axis in CLL [43]. It was 3.6 fold more toxic than CAL-101 in malignant B-CLL lymphocytes in vitro. A study on 65 B-CLL patients revealed that NVP-BKM120 was cytotoxic in 78% of the primary B-CLL lymphocytes [20].

### The roles in diffuse large B cell lymphoma

DLBCL represents the most common subtype of NHL. It accounts for 40% of newly diagnosed NHL in the world and approximately 40–50% of newly diagnosed lymphoid neoplasms in China [44,45].

Dysregulation of the PI3K/Akt/mTOR signaling pathway was observed in DLBCL [46,47]. Xu et al. investigated the activation of PI3K/Akt/mTOR signaling pathway and their clinical significance in 73 DLBCL cases [44]. Activation of this pathway was related to poor treatment response and decreased survival time in DLBCL patients treated with CHOP chemotherapy regimen (cyclophosphamide, doxorubicin, vincristine, and prednisone) but not in those treated with rituximab-CHOP (R-CHOP) [44].

Previous studies have indicated that apoptosis of DLBCL cell lines could be induced by LY294002, a pan-isoform PI3K inhibitor [22]. NVP-BEZ235 is a novel dual inhibitor of PI3K and mTOR. Concurrent inhibition of PI3K and mTOR by NVP-BEZ235 resulted in the down-regulation of Eif4e phosphorylation and MCL-1 expression. It could inhibit the proliferation of DLBCL cells via inhibiting activation of PI3K, mTORC1 and mTORC2 in both central B-cell (GCB) and activated B-cell (ABC) subtype of DLBCL [16]. But when the concentration of NVP-BEZ235 was 0.5 μM or below, the induction response of cell demise in ABC cell lines was less efficient than that in GCB cell lines.

Recent studies have highlighted that NVP-BKM120, a pan-class I inhibitor of PI3K/Akt/mTOR signaling pathway. NVP-BKM120 reduced cell proliferation and increase the apoptosis of DLBCL cells through blocking the autophagy,as well as up-regulating Puma and Bim and inhibiting anti-apoptotic Mcl-1 expression [21]. Additionally, a phase I and dose-escalation study of NVP-BKM120 provided evidence of the feasibility of PI3K inhibitors in patients with advanced solid cancers [48]. Although few of them were moved into clinical application currently, the PI3K inhibitors will bring up new therapeutic options for relapse/refractory DLBCL.

### The roles in mantle cell lymphoma

Mantle cell lymphoma (MCL) accounts for about 6% of all NHL and the median age at diagnosis is about 65. It is characterized by chromosomal translocation t(11;14)(q13;q32) resulting in over-expression of cyclin D1, which are regulated by the Akt/mTOR signaling pathway [49,50]. Despite the relatively good response to first-line chemotherapy, most of the MCL patients relapsed eventually.

Recent studies have revealed the importance of PI3K/Akt/mTOR signaling pathway and clinical application of PI3K inhibitors in MCL [51,52]. Gene expression profiling of both purified leukemic MCL cells and the naive B cells were performed through oligonucleotide microarrays [53]. 106 genes were found to be differentially expressed at least three fold in MCL cells compared to naive B cells, with 43 downregulated and 63 upregulated. Several genes relating PI3K/Akt signaling pathway were found to be aberrantly expressed in MCL cells compared with naive B cells, such as PIK3CA, PDK2, PDPK1, AKT1, RPS6KB2, FOXO3A, PPP2R2C and PDK1 [53]. Moreover, increased gene copy number ( ≥ 3) of PIK3CA were discovered in 68% of MCL cases and two MCL cell lines(Rec-1 and GRANTA-519) [23]. Mutation of PIK3CA gene resulted in constitutive activation of PI3K and the consequent activation of Akt pathway in MCL. They further investigated the apoptosis of MCL cell lines treated with LY294002. The apoptotic rates increased from 3% to 20% in GRANTA-519 cells and from 7.3% to 20% in Rec-1 cells [23].

RAD001 (everolimus), an mTOR inhibitor, could halt the translation of proteins critical for cell survival and proliferation via inhibiting mTOR phosphorylation [54]. Approximately 40 ~ 65% antiproliferative effects was found in MCL cell lines treated with single agent RAD001 compared with control groups [28]. However, NVP-BEZ235 is more effective than mTOR inhibitors (rapamycin, RAD001) in inhibiting the downstream pathway of mTOR and mediating cell death. Further analysis demonstrated that NVP-BEZ235 could lead to a dose dependent down-regulation of Mcl-1 protein while rapamycin could not [55]. Civallero et al. analyzed the inhibitory effects of NVP-BEZ235 on MCL cell lines and its effects in combination with enzastaurin, everolimus and perifosine [17]. NVP-BEZ235 induced significant increase of cell apoptosis in MCL through both intrinsic and extrinsic pathways. When combined with enzastaurin, everolimus and perifosine, the NVP-BEZ235 triggered cytotoxicity was enhanced significantly [17]. NVP-BEZ235 also showed a much stronger anti-proliferative function in MCL cells compared to single inhibitors of PI3K/mTOR, such as NVP-BKM120 and RAD001 [56]. Additionally, NVP-BEZ235 could synergistically enhance the cytotoxic function of conventional anti-tumor agents and remarkably overcome the acquired bortezomib resistance in MCL [56].

CAL-101 was reported to inhibit constitutive activation of the PI3K/Akt/mTOR pathway and exert potent antitumor effects across a wide range of B-cell malignancies [39]. Previous studies have demonstrated the functions of CAL-101 in PI3K inhibition and pro-apoptosis effect in NHL cell lines. A phase I study focused on the safety and activity of CAL-101 in patients with relapsed/refractory hematologic malignancies was carried out recently [19]. A total of 55 patients (18 MCL patients included) enrolled, CAL-101 was administered orally once or 2 times per day continuously in a 28-day cycle for up to 12 cycles. As a consequence, the overall response rate for MCL was 62% [19]. Nevertheless, GDC-0941, a dual p110α/δ inhibitor, was more active compared to CAL-101 in both MCL samples and cell lines [26].

### The roles in Burkitt leukemia/lymphoma

Burkitt leukemia/lymphoma (BL) is a highly proliferative B-cell lymphoma characterized by constitutive MYC expression [24]. In spite of current intensive, short-term chemotherapy regimens in BL treatment, less toxic and more targeted treatment strategies are still needed to improve BL prognosis, especially in high-risk and relapsed/refractory patients [57,58]. PI3K pathway acts as a vital determinant in the B cell receptor (BCR)-mediated survival signal in mature, resting B cells [59]. It has been indicated that the MYC-driven lymphoma is associated with mTOR activation and an endogenous DNA damage response transduced by PI3K-related kinase [60].

Activation of PI3K pathway has been found in BL tissues and cell lines. When treated BL cell lines with LY-294002, the phosphorylation of Akt kinase was largely diminished [24]. In drug-resistant Ramos and Daudi B-NHL cell lines, LY294002 treatment also accounted for the inhibition of Bcl-(xL) expression and sensitization to drug-induced apoptosis [61]. Our previous study also indicated the existence of PI3K/Akt/HSP70 cascade in Raji cells lines [4]. LY294002 significantly attenuated Akt activation, resulted in induced cell apoptosis and increased ADM and DDP sensitivity [4]. PI-103, a dual PI3K/mTOR inhibitor, was also associated with the caspase-dependent cleavage of PARP and inhibition of c-MYC activity in BL cells [29].

### The studies of PI3K inhibitors in T-cell lymphoma

Activation of PTEN-PI3K-Akt pathway in T-ALL has been assessed by array comparative genomic hybridization and sequence analysis [62]. Alterations of PTEN, PI3K, or Akt existed in 47.7% of total 44 cases. Moreover, patients with lymphoblasts harboring PTEN deletions at the time of diagnosis showed significantly adverse therapeutic consequences [62]. Furthermore, the PI3K transgenic mice could develop an infiltrating lymphoproliferative disorder [63]. Lymphomas (67%) and sarcomas (33%) occurred in p53 knockout mice, however, when p53 deletion was combined with PI3K activation, only lymphomas developed. In addition, PTEN, a negative regulator of PI3K pathway, showed decreased expression level in 66.7% of anaplastic large cell lymphoma (ALCL) cases [64]. And increased expression of PIK3CD gene (encoding PI3K δ isoform), was found in peripheral and cutaneous T-cell lymphoma [27].

P110a, p110h, p110g, and p110y isoforms of PI3Ks were expressed in T-ALL cell lines. A dose-dependent decrease in cell survival was obtained with p110a PI3K selective inhibitor. Nevertheless, PI-103 was more efficient in inhibiting T-ALL cell proliferation and inducing cell apoptosis than inhibitors that are selective only for PI3K (Wortmannin and LY294002) [25]. The pan-PI3K inhibitor, GDC-0941, resulted in arrest of all peripheral and cutaneous T-cell lymphoma cell lines in the G1 phase. When cooperated with MEK inhibitors, GDC-0941 showed a highly synergistic effect in enhancing cell cycle arrest in all T-cell lymphoma cell lines [27].

## Conclusions

In summary, PI3K signaling pathway was activated in both B-cell and T-cell NHL and involved in the development and progression of these diseases. The PI3K inhibitors revealed significant cytotoxicity either alone or in combination with other agents in lymphocytic cells. They have promised the breakthrough data and provided an attractive treatment option for anticancer therapeutic intervention of NHL. However, further investigations are still required to get a better understanding of the clinical benefits of PI3K inhibitors.

## Abbreviations

PI3K: Phosphatidylinositol 3-kinase; mTOR: Mammalian target of rapamycin; NHL: Non-Hodgkin lymphoma; HSP70: Heat shock protein70; BL: Burkitt leukemia/lymphoma; RTK: Receptor tyrosine kinase; PTEN: Phosphatase and tensin homolog deleted on chromosome ten; TSC2: Tuberous sclerosis complex 2; mTORC1: mTOR complex 1; 4EBP1: 4E binding protein 1; FL: Follicular lymphoma; T-ALL: T-cell acute lymphoblastic leukemia; CLL: Chronic lymphocytic leukemia; DLBCL: Diffuse large cell lymphoma; GCB: Germinal central B-cell; ABC: Activated B-cell; MCL: Mantle cell lymphoma; BCR: B cell receptor; ALCL: Anaplastic large cell lymphoma.

## Competing interests

The authors declare no competing financial interests.

## Authors’ contributions

All authors have contributed to data preparation, drafting and revising the manuscripts. All authors have read and approved the final manuscript.
